# Neither/nor: a pragmatic philosophy for oscillating between conceptual and experiential knowledge

**DOI:** 10.1057/s41599-026-06669-3

**Published:** 2026-03-13

**Authors:** Bryan Kam, Isabela Granic

**Affiliations:** 1 https://bryankam.com; 2 McMaster University, Hamilton, Canada

**Keywords:** Philosophy, Anthropology, History, Psychology, Science, technology and society

## Abstract

This paper presents “*Neither/Nor*,” a philosophical synthesis which defines conceptual and experiential modes of knowing as complementary skills which can be deliberately trained and oscillated. The paper argues that neither theory (concepts) nor practice (experience) alone can suffice for desirable outcomes in personal flourishing or scientific inquiry. Drawing from Western and Eastern philosophical traditions—from ancient skepticism and Buddhism to modern pragmatism and cognitive science—*Neither/Nor* proposes that “latent Platonism,” the unconscious preference for abstract concepts over direct experience, contributes to both personal suffering and intellectual impasses. The paper begins with a concrete example of Type I diabetes management, demonstrating the constant negotiation between abstract formulas and embodied experience required by the disease, before providing five practical principles: (1) regard concepts and experience as trainable skills; (2) commit to oscillation between skills; (3) prioritize relations and processes over objects and states; (4) embrace trial-and-error learning; and (5) employ conditional historicism over linear causal thinking. The *Neither/Nor* framework demonstrates how these principles can reduce personal suffering, enhance scientific inquiry, and provide a methodology for evaluating diverse philosophical positions pragmatically. *Neither/Nor* points towards a way of living rather than merely an abstract theory, contributing to both individual flourishing and more flexible approaches to complex societal challenges.

## Introduction

In 1986, when the first author was 21 months old, he was dying from undiagnosed Type I diabetes. Though he exhibited the classic symptoms—voracious appetite, unquenchable thirst, and constant urination—doctors missed the diagnosis because the condition was thought to occur in adolescence. His mother, after reading the Merck Manual, qualitatively matched his symptoms to diabetes, which led to his testing and diagnosis. Only then could quantitative measures—precise units of insulin—bring his blood sugar to survivable levels. The endocrinologists credited her with saving his life.

From that moment, his survival became a matter of continuous mathematical calculation. Living with insulin injections or an insulin pump requires constant computation, where even minor miscalculations risk confusion, loss of consciousness, or seizures, which he experienced throughout childhood.

How, then, do the clean medical formulas on which his life depends relate to the messy business of living his life? The abstract formulas that keep him alive are in constant tension with experience—stress, exercise, sleep, temperature, all affecting insulin’s efficacy in ways that defy simple calculation.

Philosophers have grappled since antiquity with this fundamental tension between abstract concepts and embodied experience—a question which, we argue, remains urgently relevant for addressing contemporary challenges in science, technology, and society.

This paper presents a philosophical synthesis called “*Neither/Nor*” which treats conceptual thinking and experiential knowledge as complementary skills that can be deliberately trained and alternated. More specifically, we assert that **neither** theory (concepts) **nor** practice (experience) is alone sufficient for desirable outcomes. Both are required, and practice in negation can attune us to *when* and *how* to use theory or to use practice. *Neither/Nor* provides a coherent framework for understanding the conditions for using each of these modes of knowing, separately and in combination. We propose that this approach can reduce personal suffering while providing tools to evaluate claims across disciplines. Though most of our lives may not depend so drastically on the relationship between theory and practice, learning to employ the two skilfully can allow for individual and collective flourishing.

This paper is written for an interdisciplinary readership that includes philosophers, cognitive and developmental scientists (especially those concerned with how concepts relate to experience), and educators and practitioners seeking methodological tools for reducing suffering and supporting flourishing. Our aim is not to assume prior expertise in pragmatism or other philosophical traditions, nor to address only specialists in any one domain. Instead, we write for readers who recognize that contemporary problems—scientific, personal, and societal—do not respect disciplinary boundaries and therefore require a framework which can move across them.

We have begun this paper unconventionally, with an experiential narrative, because in it we find the developmental origins for a lifelong pursuit of answers to a single question: how do abstractions relate to experience? We will return to the diabetes example throughout this paper in order to demonstrate the utility of philosophy generally, and *Neither/Nor* specifically, in everyday life and in the pursuit of scientific, academic, and practical results. This approach will illustrate our commitment to philosophy as a process of inquiry that is continuously grounded in experience and moves towards pragmatic ends.

We presuppose that philosophy functions as a way of life, concerned with flourishing and reducing suffering. We find the Greek concept of *eudaimonia*—encompassing purpose, meaning, vitality, social connection, agency, and psychological well-being—a useful way to define flourishing, though *Neither/Nor* does not prescribe a single definition and remains compatible with diverse philosophical traditions (Stoic tranquillity, Epicurean pleasure, Buddhist equanimity, Taoist *wuwei*, Pyrrhonist *ataraxia*). *Neither/Nor* is fundamentally a methodology, not a dogmatic prescription for how to define flourishing.

In this paper, we have three objectives. First, we present *Neither/Nor*, a philosophical framework for utilizing two fundamental forms of knowledge: conceptual and experiential. We argue that the naive belief that concepts are primary and embodied experience secondary produces systematic suffering. We call this view “latent Platonism.” *Neither/Nor* functions as (1) a philosophical framework; (2) a methodology for healthy inquiry that evaluates contributions across disciplines; and (3) a set of practical principles for individual and collective flourishing. Our second objective is to discuss the lineages contributing to Neither/Nor, covering key insights from Western and Eastern thinkers from antiquity to the present. This approach provides new interpretations of ancient texts and methodologies for evaluating diverse philosophies when confronted with real-world problems. Our final objective is to present five central principles of *Neither/Nor*, and to suggest their practical applications to both personal suffering and scientific inquiry.

## Philosophy and latent Platonism

The first author’s survival with diabetes depends on mathematics, formulas, and abstraction, day and night, to maintain a survivable blood sugar level: eating 40 g of carbohydrates requires five units of insulin. Yet this neat calculation becomes complex when blood sugar levels vary, during exercise, fasting, stress, inadequate sleep, temperature changes, or insulin pump malfunctions—all affecting insulin’s efficacy. While theoretical models and formulas developed by researchers are indispensable, the messiness of daily life challenges these abstractions. This negotiation between abstract and experiential knowing extends beyond diabetes—it is an essential tension which manifests in daily life at every scale, whose examination dates back to ancient philosophy.

But why revisit ancient philosophy? Because philosophical assumptions shape how we live, whether we recognize them or not. Those unaware of philosophy aren’t exempt from it—they simply operate unconsciously within inherited, unexamined philosophical frameworks.

*“[T]hose who are in ideology believe themselves by definition outside ideology: one of the effects of ideology is the practical denegation of the ideological character of ideology by ideology: ideology never says, ‘I am ideological.*’ (Althusser 1971)

We claim that Western philosophical defaults—unconsciously adopted—increase suffering for individuals and society. In contrast, many ancient and non-Western philosophies avoid the assumptions we will label “latent Platonism.” Despite millennia of original perspectives from these philosophical minorities (Alcoff 2006; Mills 1997; Van Norden 2017; Salami 2020), Western philosophy largely ignores traditions that do not prioritize conceptual, abstract, and speculative views on non-evident matters.

Western philosophical assumptions include the notions that concepts are innate, that conceptual constructs supersede experience, that nature itself conforms to concepts, and that nature therefore follows logic and simplistic cause-and-effect patterns. Such assumptions correspond to what the Buddha termed “speculative views,” Greek skeptics called dogmatic *doxai* (“beliefs”), and von Bertalanffy (2015) labeled “mechanistic” thinking. We suggest that many hold an implicit preference for such views, which we will term “latent Platonism.”

Platonism posits transcendent, eternal “forms” which exist independently of the sensible world, and which provide meaning and value to reality (Plato 1997, bk. V–VII; Blumenthal and Armstrong 2024; A. Miller 2024). Though most modern thinkers would reject Platonism stated this way, our everyday language betrays implicit commitments to abstract concepts. When we attribute agency or mind-independent existence to concepts like “social media” or “capitalism,” we unreflectively reify abstractions without clear referents. We do not argue against such usage, but observe that it reveals problematic assumptions. Because such usage is often unconscious and can contradict our stated beliefs, we call this kind of Platonism “latent.”

Latent Platonism shapes our fundamental assumptions about reality and truth. It leads us to treat nature as inherently conceptual and amenable to analysis, and often leads to a “correspondence theory of truth” (David 2022). Such a theory asserts or assumes a static objective reality which is mind-independent, and measures our endeavors by the degree to which they “correspond” to this pre-existing reality.

The correspondence theory, since Plato and Aristotle, effectively transformed abstract concepts into object-like entities and propositions into facts that can be manipulated apart from their social origins. Dewey termed this error the “philosophic fallacy” (Dewey 1925, 29)—treating the results of inquiry as if they existed prior to the inquiry itself. There are alternative theories of truth, including Peirce’s convergence theory (Charles S. Peirce 1878) and James’s pragmatic theory (James 1907), which emphasize truth as a process rather than static correspondence. However, correspondence theory remains the dominant and often unconscious framework in many domains, and its treatment of experience as “corresponding” to a reified conceptual reality exemplifies the latent Platonism we address. Our focus is narrower: we critique correspondence theory specifically because our core premise here is that it functions as an unexamined assumption that contributes to the patterns of suffering and intellectual rigidity we describe. Providing a comparative full treatment of truth theories is beyond our current scope.

While latent Platonism operates as an unconscious philosophical stance, its effects on personal experience are concrete and observable. The move from treating concepts as provisional tools to treating them as objective realities creates specific forms of suffering through what we might call “concept misuse.” The first author’s experience with doctors who were charged with helping him manage his diabetes illustrates how reifying medical formulas as fixed rules (treating “40 g carbohydrates = 5 units insulin” as an absolute truth) could regularly create suffering; that is, his own frightening experiences with spiking blood sugar levels would contradict the abstract, ideal version of what the doctors expected from applying a “rigorous” formula—which, for a myriad of “life” reasons, might fail. The attitude in the medical community that if something went wrong, it must be the patient’s error rather than the insufficiency of the approach, while far from universal among compassionate endocrinologists, is well-documented. For example, research on healthcare provider bias in obesity-related diseases shows that physicians often attribute poor outcomes to patients’ lack of willpower or motivation rather than examining treatment protocol limitations (Puhl and Heuer 2009).

To relieve suffering, we do not advocate the simple opposite of conceptual reasoning, claiming that experience trumps concepts or that nature is not subject to logic. This would merely substitute one “speculative view” for another. Instead, we treat both perspectives as skills to be deliberately oscillated. Oscillation, in our sense, means fluctuation and deliberate alternation between states, rather than periodic motion; we avoid “alternation” itself because it implies a binary rather than a spectrum. We view the relationship between concepts and experience as being two interdependent and spectrum-like skills rather than binary, and yet by recognizing such a division, we argue that the skills can be isolated and trained separately.

## Why do we need further philosophy?

We need philosophy, first, because philosophy has led us here; our current crises are rooted in ideologies inherited from past philosophy (Tweedale 2023). We face a critical transition period (Brozović 2023) driven by global internet participation, ubiquitous mobile devices, and artificial intelligence, comparable to the Renaissance or Industrial Revolution. Communications technologies create feedback loops that destabilize complex systems, generating urgent challenges: mental health crises (Ernst et al. 2022; Surkalim et al. 2022), political polarization (Iyengar et al. 2019), climate change (Ripple et al. 2024), and eroding trust in institutions (Valgarðsson et al. 2025). The severity of these challenges has prompted unprecedented responses, such as the joint statement from over 200 health journal editors calling for emergency climate action through “collective action” and “global coordination” (Atwoli et al. 2021). The Intergovernmental Panel on Climate Change has called for fundamental societal transformations to achieve climate-resilient development (Schipper et al. 2022).

These personal, collective, and societal challenges require fundamental re-examination of the assumptions undergirding entire domains. Such re-imagination demands a cross-disciplinary philosophical framework that utilizes diverse perspectives while preserving their distinctive contributions. *Neither/Nor* provides a mediating role and *tools for healthy inquiry* across these domains (for pragmatist approaches as playing a mediating role, see Pihlström 2023). By “healthy inquiry,” we mean investigation that enhances functioning—whether of an individual, a discipline, or a community. Following Spinoza’s health model of morality, we understand “what is healthy for inquiry” not as a fixed moral category but as what is useful for enhancing one’s capacity to act and understand (Spinoza 1985, pt. IV pref). Healthy inquiry thus avoids both the dogmatic rigidity of latent Platonism and the paralyzing doubt of excessive skepticism.

Our approach aligns with James’s pragmatic view (1907) that healthy inquiry enables successful action by producing concepts that “work” in experience. Healthy inquiry, then, is inquiry that leads to increased capacity for both individuals and disciplines—related to the reduction of personal suffering and consequent flourishing we addressed in the previous section. What enhances functioning is context-dependent. This aligns with Deleuze and Guattari’s vision of philosophy as “the art of forming, inventing and fabricating concepts” (Deleuze and Guattari 1994, 2) that are necessarily “connected to problems without which they would have no meaning” (16).

## Philosophies as ways of life

*Neither/Nor* is rooted in ancient wisdom. We draw from specific insights across Western and Eastern traditions while remaining committed to philosophy as a way of life, enacted socially in the world. This approach keeps philosophy tightly tethered to everyday problems of personal and collective suffering, making it useful and relevant for living rather than remaining abstract.

*Thus there is here supplied, I think, a first-rate test of the value of any philosophy which is offered us: Does it end in conclusions which, when they are referred back to ordinary life-experiences and their predicaments, render them more significant, more luminous to us, and make our dealings with them more fruitful?* (Dewey 1925, 7)

Wittgenstein wrote that “to imagine a language means to imagine a form of life” (Wittgenstein 2008, 7e). We liken philosophical texts to soil—like soil, they comprise the concentrated remains of former ways of living. Their words, once living and enlivening, become like bones or stones: durable artifacts that hint at how humans once lived their lives. Philosophy condenses life into language, preserving traces of past ways of living.

Even extinct philosophies can provide fertile soil for new forms of life and foundations for sciences—the European Scientific Revolution itself depended on ancient Greek works preserved through Islamic translations (Cohen 2015; Grant 2008). A piece of past writing, as a relic, hints at what tools might revive the older ways of life. To read ancient philosophy is to enter this rich reliquary. Ancient philosophy’s age is its strength; it offers resilient, durable, and tested wisdom for contemporary challenges. While we should certainly explore new thinking, untrammeled by tradition, most new philosophies will fail, whereas antiquity’s approaches once functioned effectively—often for now-neglected centuries or millennia. Foregoing this hard-won wisdom isn’t merely disrespectful, but dangerous. Understanding the past is essential to navigating the future.

Ancient Greek philosophy was, as Hadot (1995) has shown, primarily about living, not merely thinking. *Neither/Nor* extends this perspective beyond specific schools to all worldviews, however theoretical they appear: not just “Stoicism as a way of life” but “Mathematical realism as a way of life” or “Scientific materialism as a way of life.” Our preference for “way of life” over “school of thought” has practical consequences. As Western philosophy has become increasingly abstract, it’s crucial to examine the social origins of thinking itself (see “Conditional historicism” below). Although space limitations preclude us from further discussion of the social nature of knowledge, readers are referred to work by Dewey (1938, ch 24) and Peirce (1878); for more on pragmatism as a way of life, see Putnam and Putnam (2017) and Shusterman (2016).

While searching for patterns and laws remains essential to science, we suggest that finding some final, complete set of rules is neither necessary nor possible. This is because we inhabit an ever-changing world requiring constant adaptation. These new conditions demand a new language appropriate to our current circumstances. Rather than merely rehearsing ancient conceptual debates, we assert that ancient philosophies have no single meaning to recover—though the attempt to understand them on their own terms and in their original context remains invaluable in developing our own approach. Instead, we need to understand the questions they asked and how they set about answering them, using their investigative approach as a model for our modern investigation. The results must then be put into practice. Answers are never final; the best ones suggest next steps—new experiments to run and questions to ask.

With modern uncertainty mounting on multiple fronts, ancient philosophy is more relevant than ever. The foundationalist projects of the early 20th century, which sought to ground society and science in certainty, have failed (Quine 1969). This has led thinkers to a false dichotomy between dogmatism and relativism. Those craving certainty cling to “scientism” (Beale and Kidd 2017) or regress to religious dogma, ignoring inconvenient facts. Despite perennial paternalistic fears of nihilism (Jacobi 1995), few embody it beyond intellectual posturing. But whenever theories of truth are challenged, we observe that accusations of nihilism and relativism arise, reinforcing this false dichotomy.

*Neither/Nor* avoids both dogmatism and relativism, comforting itself neither with fixed certainty nor with fixed uncertainty. We privilege neither concepts nor experience. Both action and knowledge of history are needed. To gloss Kant (1998, A51/B75): to study history without taking action is empty, and to take action without studying history is blind. We must learn from history’s best thinkers while examining their axioms and background assumptions to address today’s problems at their origins. This oscillation between theory and practice—which we call the “healthy mode of inquiry”—avoids the twin pitfalls of dogma and doubt, reducing suffering at individual and collective levels.

## Applied to personal suffering

Personal suffering has motivated philosophy across traditions: the Buddha taught “suffering and its cessation” (MN 22, Ñāṇamoli and Bodhi 1995, 234), while Epicurus sought freedom from “pain and fear” (Diogenes Laertius 1959, 653). *Neither/Nor* takes inspiration from these ancient traditions. To demonstrate their contemporary relevance, we first define suffering and ground it empirically.

Epidemiological evidence reveals a crisis in human suffering. A recent meta-analysis showed adolescent depression at 24.5% globally—double pre-2010 rates (Shorey et al. 2022) and anxiety disorders rising annually among U.S. youth (Parodi et al. 2022). Depression is now a leading cause of disability for those under 25 (Gore et al. 2011), with even mild symptoms predicting academic struggles and substance abuse (Wesselhoeft et al. 2013).

Mortality data underscores this severity of suffering, with youth suicides increasing 19% over two decades (Naghavi 2019). Suicide now exceeds deaths from all infectious diseases combined for youth. Despite cultural reporting variations, indicators of loneliness and self-harm have risen across countries (Murthy 2020; World Health Organization 2022), suggesting a global crisis in personal suffering.

We argue that latent Platonism and concept misuse contribute to this trend. The concepts embedded in consumerist values correlate with declining mental health across cultures (Kasser 2016), while eroding support systems compound this crisis, leading to widespread loss of meaning (Eckersley 2006).

To give an example of how concepts can cause suffering, consider someone who struggles with the concept of “authenticity.” They believe there must exist a “true self” that can be discovered or expressed, and that their current way of being does not align with this true self. This belief (a form of latent Platonism about identity) generates distress by generating a perpetual gap between experience and an impossible ideal and by making every action suspect while simultaneously narrowing behavioral options through avoidance of anything that feels “inauthentic.” We will discuss further problems arising from the stable identity view under “Relations and processes,” below.

Our claim is that the suffering generated by rigid concepts is structural, not tied to any particular type of concept. No lived experience can definitively confirm alignment with a reified concept, because direct experience always exceeds conceptual categories. Similar patterns of suffering emerge around reified concepts of romantic love (“finding the one”), career fulfilment (“finding my calling”), or emotional states (“overcoming my anxiety” where anxiety is treated as a discrete entity to be eliminated). In each case, latent Platonism transforms what could be flexible, context-sensitive descriptions of patterns into rigid ontological commitments that experience can never fully satisfy. We argue that because concepts can act as an anesthetic, and give the impression of “object-like” stability, tractability, and control, such naming can be adaptive in the short run.

In the long run, however, latent Platonism ultimately worsens suffering by devaluing experience and impeding action. Believing, for example, that “injustice” categorically exists (or does not) represents a metaphysical position, often adopted unwittingly. From this fixed categorical stance arises suffering. We don’t deny injustices—our concern is with the *additional* suffering caused by categorical judgment. Our critique extends to any system for determining what is intrinsically “good” or “bad,” which diverts individuals from investigating their values themselves. Nietzsche recognized the dangers of treating “good” and “evil” as an innate pair (Nietzsche 2003; Nietzsche 2013). Before him (~200 CE), Greek Pyrrhonists advised that “those who make no determination about what is good and bad by nature neither avoid nor pursue anything with intensity” (Sextus Empiricus 1994) and thereby cease to suffer.

Extant data from intervention and experimental studies demonstrate how the *Neither/Nor* framework can address suffering through two complementary pathways. The first pathway reduces suffering by dissolving problems created through conceptual reification. Research on cognitive defusion within Acceptance and Commitment Therapy (ACT) demonstrates that distress decreases when individuals learn to observe thoughts as mental events rather than as absolute truths (Hayes et al. 2006; Levin et al. 2012). Similarly, metacognitive therapy (MCT) research shows that shifting from engagement with thought content to awareness of thinking processes reduces rumination and worry (Wells 2009). Studies also show that psychological flexibility (the ability to not get stuck in rigid thought patterns) is strongly associated with better mental health and that inflexibility (rigidity in thoughts, emotions, and behavior) is linked to a broad range of psychopathology across anxiety, depression, and other disorders (Kashdan and Rottenberg 2010). The relief comes not from finding better concepts but from recognizing that the search itself was predicated on treating provisional descriptions as metaphysically stable truths.

Dewey anticipated these patterns in *The Quest for Certainty*, arguing that “the quest for certainty is a quest for a peace which is assured, an object which is unqualified by risk and the shadow of fear which action casts” (1929a, 7). He noted that “‘Safety first’ has played a large role in effecting a preference for knowing over doing and making” (6). This analysis aligns with our view of latent Platonism: “the quest for complete certainty can be fulfilled in pure knowing alone. Such is the verdict of our most enduring philosophic tradition” (7). Dewey’s framework thus illuminates why concepts can function as an anesthetic—providing the “object-like” stability that action in an uncertain world cannot guarantee.

The second pathway enables flourishing by opening possibilities previously foreclosed by conceptual rigidity. This shift moves one from a mode of *discovery* to a mode of *creativity*—recognizing they are partly authoring aspects of their lives through their choices rather than discovering pre-existing truths about themselves. Self-determination theory shows that autonomy is a fundamental psychological need associated with well-being and vitality (Ryan and Deci 2000; 2017), while growth mindset research shows that believing capacities can be developed predicts greater achievement and resilience (Dweck 2016; Yeager and Dweck 2012). Studies on meaning-making show that active construction of meaning predicts greater well-being following adversity (Park 2010). Most fundamentally, provisional use of concepts allows greater attention to immediate experience itself. Meta-analytic research demonstrates that mindfulness, which partially suspends concepts in favor of embodied experience, is associated with reduced symptoms across disorders and increased well-being (Goyal et al. 2014; Khoury et al. 2013). These positive outcomes represent distinctive ways of thriving that emerge from applying *Neither/Nor* methods in everyday life.

## Applied to scientific and philosophical inquiry

One of our core assertions is that the methods effective for reducing personal suffering also apply to scientific inquiry and philosophical conflicts. *Neither/Nor* does not simply revive older philosophical traditions or add another position to the canon. It helps individuals and cultures experiment with different frameworks by way of operationalizing a methodology for healthy inquiry that avoids both the rigidity of latent Platonism and the paralysis of excessive skepticism. In this sense, the framework provides cross-disciplinary tools that make earlier philosophical insights usable as living methods, rather than static metaphysical claims. The patterns we highlight appear in both the Buddhist account of suffering and Kuhn’s account of scientific revolutions (see “Conditional Historicism”), suggesting that inquiry, at any scale, follows similar structural dynamics—as Dewey separately claimed (Dewey 1938, ch 6).

We assert specifically that Buddhism, one of the most detailed analyses of how experience works, aligns with Thomas Kuhn’s detailed analysis of how group conceptual change works. The alignment is mutually illuminating and can be operationalized. First, we note the similarities. Just as Kuhnian scientific revolutions are precipitated by accumulated anomalies that cannot be resolved within the existing paradigm (Kuhn 2012), the Buddhist path begins with *dukkha*—the persistent unsatisfactoriness of experience organized by dependent arising (SN 56.11 Bodhi 2000, 1843). In both cases, the crisis reveals that the conceptual framework itself has become an obstacle. Kuhn’s scientists must reverse direction methodologically, examining foundational assumptions which normal science treats as settled; Buddhist practitioners reverse dependent arising’s links, tracing back through the causal conditions that construct the sense of self (Ñāṇananda 2012). Both practices reveal conditionality where causal necessity seemed obvious: what appeared as the structure of reality (Newtonian space, the autonomous self) becomes visible as a contingent framework maintained by specific historical or experiential conditions. The change in both cases by this backwards traversal is a revolutionary shift in perspective. The parallel lies not in content but in structure—both describe how frameworks that organize inquiry or experience can generate intractable problems requiring not optimization but revolutionary transformation through examining the generative conditions themselves.

Without strictly adhering to Kuhn’s definition of crisis, we find his analysis illuminates how disciplines seek new models amid uncertainty. We observe uncertainty within disciplines themselves—for example, psychology’s replication crisis (Open Science Collaboration 2015)—and argue that during such periods, philosophy has the opportunity to provide methodologies, synthesis (Schliesser 2019), and connective tissue across disciplines.

As a methodology for healthy inquiry, *Neither/Nor* suggests specific skills and strategies by which scientific paradigms can be evaluated, integrated, or set aside. Just as breakthroughs spur science toward new horizons, philosophy constantly evolves to support scientific inquiry. This creates a productive oscillation: philosophers propose conceptual frameworks, scientists collect data and run experiments (which take place in experience) and propose theories of their own, and philosophers compare the theories across disciplines.

Healthy inquiry enhances a discipline’s vitality—mirroring our approach to reducing personal suffering and allowing flourishing, by oscillation of methods. We use this method to provide a postscript to past schools of thought, which we categorize as tools to be employed by their dominant tendencies. Viewing past philosophical and scientific positions as trainable skills allows us to learn from different schools with incompatible axioms, picking up and setting down their tools as appropriate.

Our approach advocates active investigation over static conceptual frameworks, avoiding both excessive theorizing (naive rationalism, as seen in “scientism”) and wholesale theory rejection (naive irrationalism, as seen both in anti-rationalist conspiracy views, religious fundamentalism, and the tech sector’s “move fast and break things”). Instead, we propose a pragmatism influenced by Eastern philosophy, where inquiry yields multiple perspectives on how to proceed.

In the next section, we’ll show how *Neither/Nor* applies more specifically. We outline five core principles underlying our approach to healthy inquiry—principles equally relevant to addressing personal suffering and investigating scientific and philosophical challenges.

## Five principles of *Neither/Nor*

### Two trainable skills

We learn about and act within the world in two inseparable ways: through concepts (reason) and through perception/action (experience). Reason uses language, abstraction, and mathematics—clean and orderly concepts. Experience comes from perception and action—encounters with what Nietzsche (2000) calls the “incompletely intelligible everyday world,” and what Dewey called “non-reflectional experience” (1916a, 4). Though often in tension, these modes are never entirely separable: we can’t learn concepts without perceiving, nor experience life without preconceptions.

This distinction between modes of knowing has appeared throughout the Western tradition. Plato (~369 BCE) separated “perception” from “true judgment” in the *Theaetetus* (1992), while Aristotle (~350 BCE) distinguished “perception” from “reason” in *De Anima* (2016, III.4). Buddhist philosopher Dignāga (520 CE) contrasted direct perception with inference (Dignāga 1968). Medieval thinkers like Al-Farabi (~950), Avicenna (1026), Averroes (~1198), and later thinkers in the West, like Aquinas (~1274), and Scotus (~1302), maintained the same distinction (e.g., Aquinas 1920, Ia q. 78 a. 1; Duns Scotus 2024, II d.2 2 q.2 n.321).

Early Modern philosophers have been divided into “rationalists” like Leibniz and Descartes (who emphasized concepts), and “empiricists” like Locke and Hume (who emphasized the senses). Yet thinkers across the divide made the same distinction. Hume divided experiential “impressions” from conceptual “ideas” (1739), while Descartes divided “sensation” from “intellection” (1996). Kant emphasized their interdependence: “The understanding is not capable of intuiting anything and the senses are not capable of thinking anything” (1998, A15/B29). Similar distinctions appear in Locke, Berkeley, and Mill, and in the 20th century, in Russell, Moore, and Ayer (Ayer 1979). Russell distinguished “sense data” from “thinking,” and made a related distinction between “knowledge by acquaintance” (experience) and “knowledge by description” (reason; Russell 1911). This distinction persists in contemporary philosophy of mind, between experiential “qualia” and conceptual “propositional attitudes” (Chalmers 1996).

We emphasize “Neither/Nor” over “Both/And” for a crucial methodological reason: combining the two skills into a single unified process—as “Both/And” approaches do—tends to obscure rather than reveal the specific qualities of each skill. The first stage, we argue, requires *isolating* each skill through deliberate practice and developing the ability to recognize and reverse each skill (what we call “negation”), *before* any subsequent combination. By “negation,” we mean the capacity to actively suspend or reverse one’s active skill—for example, stopping conceptual analysis to return to direct experience, or interrupting habitual action to engage in reflection. This isolation and negation must precede integration. “Neither/Nor” thus precedes “Both/And” approaches, which, if adopted too soon, prevent the isolation required to understand each skill on its own terms. Only after developing facility with isolation and negation can one skilfully combine the skills in a unified process.

Ancient thinkers dichotomized the positions, whereas modern thinkers view them as complementary. We side with the complementary view—neither concepts nor experience are simply “given” (Sellars 1991)—but more importantly, we assert the divide neither ontologically nor epistemologically, but methodologically. Rather than debating which is better, we argue that separating them allows each to be trained independently. To make a physical analogy, all physical motion involves both the muscular and the cardiovascular system. But it is still possible to undertake strength training or cardiovascular training, which emphasizes one of the two. A similar dynamic characterizes the relationship between cognition and emotion (James 1884; Lang 1994; Schachter and Singer 1962). Each can be emphasized separately while recognizing their fundamental interdependence.

Kant emphasized the distinctness of these two faculties (the **understanding**, which deals with concepts, and **sensibility**, which deals with the senses or “intuitions”), linking the two faculties with a third—the **imagination**—with his doctrine called the “**schematism**” (Kant 1998, A137–A147/B176–B187) Because the schematism posits a conceptual a priori set of rules, many thinkers have found it unsatisfactory or insufficiently articulated—including, for different reasons, Schopenhauer (1969, app. “Criticism of the Kantian Philosophy”), Dewey (1925, ch 8; 1929a, ch 4; 1938, ch 25), and Heidegger (1997). Pragmatist thinkers sought to reject the dichotomy between concepts and perception under the single process of “inquiry.” The American pragmatist philosopher Charles Sanders Peirce established the pragmatist framework for inquiry in “The Fixation of Belief” (1877). He argued that reasoning (“drawing inferences”) itself is a “long and difficult art,” and that inquiry originates not in artificial conceptual questions, but in a “real and living doubt.” This genuine doubt is an “uneasy and dissatisfied state” (not unlike Buddhist *dukkha*) from which we struggle to free ourselves and pass into belief. The “irritation of doubt” drives us through “inquiry” and toward “belief,” a settled disposition that guides our responses to experience. This oscillation between doubt and settled belief—the engine of inquiry—prefigures our oscillation between experiential and conceptual knowledge. As we do, Peirce rejected both the “method of tenacity” (clinging to concepts regardless of experience) and atomistic empiricism, advocating instead for a self-correcting scientific method grounded in community verification.

John Dewey built on Peirce’s process of inquiry, adding aspects which align with our approach. Like Dewey, we treat knowing as an active, developmental process rather than a static faculty. We share Dewey’s insistence that inquiry is rooted in lived experience, arises in response to indeterminate situations, and ultimately serves the pragmatic aim of improving the experience of life. Dewey’s pattern of inquiry, beginning with felt tension and ending with qualitative resolution (1938, 6), maps onto our oscillation between conceptual and experiential modes. We agree that thinking is not a passive mental event but a practical, embodied response, involving hands, tools, environments, and social relations (1916a, 14). And we likewise functionally divide theorizing from practice, in a similar way to how Dewey made a “functional division of labor” (Dewey 1938, ch 25) between “analysis” (breaking down a tensional situation into parts) and “synthesis” (recombining these parts into new functional relationships) in the “process of inquiry” for the purpose of examining each. Finally, like Dewey, we reject the empiricist/rationalist split as a false dichotomy arising from isolating phases of a single unfolding process; instead, we treat both modes as essential to intelligent action and flourishing.

Where we diverge from Dewey is in treating the conceptual and experiential not as a single unified process (which he terms “intelligence”) but as two separate, trainable skills, each with its own history, in both phylogeny and ontogeny. Evolutionary and developmental evidence support the distinction between these skills. Perception precedes abstract concepts in both species and individual development. Evolutionarily, even single-celled organisms show sensory abilities (Lyon 2015) and some demonstrate learning (Dussutour 2021), though only in direct interaction with an environment. Chimpanzees show great intelligence in reasoning with objects but limitations in abstract reasoning (Call and Tomasello 2008).

Research on infant cognitive development likewise shows that perceptual and sensorimotor abilities emerge many months before symbolic or conceptual reasoning. Infants demonstrate sophisticated perceptual discrimination and object permanence by 4-6 months (Baillargeon 1987), while the capacity for symbolic representation and abstract categorization emerges substantially later, around 18–24 months, with the development of language and pretend play (DeLoache 2004). These studies show that the capacity for experiential engagement precedes conceptual abstraction, supporting their treatment as separable, trainable skills.

Dewey combines these capacities into a single functional process. We, by contrast, recognize the value of Kant’s division and the centrality of the schematism, and the need for an explanation of how the two relate, which is obscured rather than revealed when collapsed into a single process. Dewey avoids these distinctions to prevent the reification of “thought” into “a substantial entity” and to resist treating reflective operations as “an entity of a wholesale nature called Thought or Reason” (Dewey 1938, 522–523). He argues that positing the “antecedent existence of an a priori rational world puts the cart before the horse” and that such assumptions require “drawing a hard and fast metaphysical line” between the senses and reality (524).

But it’s precisely because Dewey’s “intelligence” is an “achievement, not an original endowment” (1925, ch 9; 1929a, ch 2) that we argue that its constituent skills require explicit, separate training if they are to improve as a unified whole. Drawing on empirical research on expertise, deliberate practice, and developmental psychology, *Neither/Nor* operationalizes Dewey’s “functional division of labor” (1938, 509) not merely for analysis but for pedagogy and for practice: to teach individuals how to perform the process of inquiry that Dewey describes. Critically, this methodological separation serves a pedagogical purpose: by training each skill independently, we develop the capacity to overcome the very dualism that separation initially requires. The separation is instrumental and temporary—a means to develop facility with oscillation, not an endorsement of dualism as a metaphysical truth.

This operational focus aligns with Dewey’s own insistence that pragmatist concepts must be “operationally instituted” (Logic, Preface). Expertise, furthermore, can be seen as a process of moving from conceptual (rules-based) to experiential (intuition; see Dreyfus and Dreyfus 2005). This model supports our operational claim—and our position is not passively Kantian, but actively operational and intended for practitioners. Where Dewey emphasizes the functional unity of intelligent inquiry, *Neither/Nor* focuses on the training regime that allows the improvement of this unity, rendering Dewey’s philosophical insights more actionable, measurable, developmentally applicable, and testable. As Nietzsche observed, “The most valuable insights are arrived at last; but the most valuable insights are methods” (1968, 261, §469). Dewey, too, emphasized operationalization of method, testing conceptual hypotheses with experimental action to see if the concepts are valid (Dewey 1916a, 10–12). The method of inquiry (i.e., how one relates concepts to experience) determines both the emergence of suffering and the possibility of its alleviation.

For our operational purposes, then, let us first examine the conceptual skill (we will treat the experiential skill in the next section on oscillation). Humans uniquely rely on abstract concepts (“the economy”, “the Big Bang”). Since Aristotle (2016; *De Anima* II.3), abstract reasoning has been claimed to be a major distinguishing feature of humans. While other animals use concepts (Glock 2000), human life distinctively depends on abstractions that shape perception, thought, and action.

Human concepts build upon evolutionarily older capacities. In development, too, infants sense before speaking, speak before reasoning, and grasp Aristotelian particulars before universals. We share neural mechanisms for pre-linguistic understanding with other primates, forming the foundation for language development both individually and evolutionarily (Rizzolatti and Arbib 1998). As Vygotsky and Luria note, “speech lifts action to its highest stage, action that was previously independent of it” (1994, 169).

While reasoning has clear benefits, it also produces and perpetuates problems. Buddhism identifies reified “self-view” as a source of suffering (SN 22:59, Bodhi 2000, 901), tracing a path from conceptual proliferation (*papañca*) through thinking and desire to suffering (DN 21, Walshe 1995, 263). This conceptual proliferation drives both interpersonal conflict (Sn 4:11, Bodhi 2017, 1135) and internal rumination (MN 18, Ñāṇamoli and Bodhi 1995, 202).

Using concepts wisely requires awareness that they differ from direct experience. While deductive reasoning can be infallible given correct premises (Hume 1766, sec 1.4), we must take empirical experience seriously, despite its uncertainty.

Contemporary research links mental health problems to concept misuse and excessive reasoning. Depression and anxiety are amplified by abstract thinking patterns like rumination (McLaughlin and Nolen-Hoeksema 2011) and catastrophizing (Brosschot et al. 2005). Effective interventions since Buddhism have focused on altering thought patterns by first changing behavior (experience). Each skill has costs and benefits. *Neither/Nor* advocates for training these as independent skills and deliberately oscillating between them.

Distinguishing these two modes of knowing can have important practical effects. In medical decision-making, where diagnostic accuracy is critical, research consistently demonstrates that effective clinicians require the use of both skills: they recognize patterns through accumulated clinical experience while also engaging in deliberate analytical reasoning when cases are complex or unfamiliar (Eva 2005; Mamede et al. 2010; Norman et al. 2017). Diagnostic errors have been traced not to the absence of either mode, but to insufficient development of one or both—suggesting that expertise requires cultivating each capacity, not privileging one over the other.

### Commitment to oscillation

A core claim of *Neither/Nor* is that isolating and alternately practicing conceptual and experiential modes of knowing provides us with a healthy mode of inquiry, and consequently a better means to navigate life. Though neither exists in pure form, individuals can strategically employ one skill over the other, gleaning benefits from each. This requires not just moving between theory and practice, but also cultivating a tolerance for uncertainty through periods of active suspension—deliberately neither thinking conceptually nor acting experientially, but simply waiting and observing. We call this deliberate alternation, with periods of patient waiting, “oscillation.”

Oscillation is not periodic or mechanical like a pendulum’s regular swing. Rather, it is context-dependent and responsive: one switches between skills (or suspends both) based on situational demands, not on a fixed schedule. This oscillation between stable concepts and unstable experience reflects Dewey’s view of existence’s dual character as both “stable” and “precarious” (1925, 41)—inquiry arises in response to problematic situations which emerge from the “intricate mix of the stable and the precarious, the fixed and the unpredictably new” (59). In the long run, neither reason nor experience alone enables us to change ourselves or the world. By oscillating between the two—and by practicing suspension—we learn what each feels like and become more adept at using both. This is not like balancing a scale, but like an organism in motion, balancing in relation to the environment and its intentions. Flexibility in oscillation can be taught, learned, and practiced. Knowing when to use language (concepts), when to experiment (experience), and when to suspend both is key to our practical approach. Navigating life well requires making this oscillation conscious and deliberate. This practice demands self-awareness and the courage to switch skills—or to suspend activity entirely—even when counterintuitive.

To return to the diabetes example, dosing insulin requires concepts like carbohydrates, but its effects are always experiential. The result is not random; there are bounds. But rarely is it an exact science. Similarly, baking a cake requires precise measurements (conceptual) alongside practical procedures (experiential). The pattern repeats fractally—measuring flour involves both numerical quantification and embodied action. These cases begin with concepts and move to experience. Other endeavors may reverse this trajectory; to write a memoir, for example, moves from experience to concepts.

While oscillations between theory and practice are clear in these examples, other domains require training to recognize which skill predominates. The diabetic needs abstraction to survive, yet must constantly adapt formulas to life’s messiness. The solution lies neither in rigid adherence to abstractions nor in impulsive improvisation. Instead, the goal is to become sensitized to overreliance on either skill, recognizing when to switch modes or suspend activity, thereby allowing theory to inform practice and practice to reform theory.

Modern education results in biased training, either theoretically or in some domain of action. Within disciplines, an antipathy or friendly rivalry often arises between those who specialize in the opposite strategy, for example—between pure and applied mathematicians (Hardy 2019), between theoretical and experimental physicists (Hahn 2019), and between theory-driven psychologists and clinical practitioners (Kazdin 2008). Such specialization supports our view that both skills are trainable. *Neither/Nor*, however, emphasizes the practical skill, eschewing intellectual foxholes, if only because the rational skill is already highly valued.

According to our approach, there are common failures from overreliance on either skill. An overreliance on concepts leads to dogmatism, rigidity, blindness to counter-evidence, and doubling down on failing strategies, while an overreliance on experience can paralyze action through excessive doubt, preventing the provisional agreements needed for collective progress.

Modern education teaches us to value conceptual frameworks and to set goals. To a lesser degree, it teaches us to take action and to test evidence. It rarely teaches systematic deconstruction of systems and their axioms. And it never explicitly teaches deliberate alternation between thinking and acting, with long periods of active suspension, training tolerance for uncertainty while neither thinking nor acting. Yet practices which implicitly oscillate—from vipassana meditation to interval training to dialogical therapies—have shown promise in addressing depression, which we link to rigid conceptual habits (e.g., Beck et al. 2024).

A final practical lesson from this principle of *Neither/Nor*: we advocate starting with the opposite skill from the one you seek to advance. To learn meditation (experiential), begin with books or teachers (conceptual). To write theory (conceptual), begin with stories or experiments (experiential). This paper demonstrates this principle—starting with lived experience before moving to theory—and ultimately returning to practice.

Eva (2005) examines how expert clinicians navigate diagnostic reasoning and demonstrates that both analytic (deliberate, rule-based) and non-analytic (intuitive, pattern-recognition) processes contribute to diagnostic accuracy. He argues that these two forms of reasoning work together interactively rather than sequentially, with “both forms of processing contribute to the final decisions reached in all cases.” Eva emphasizes that clinical teachers should help students develop both capacities, enabling them to draw upon multiple reasoning strategies depending on the context. Expertise involves having a rich repertoire of approaches available—often deployed unconsciously—rather than mastering any single mode of reasoning. This suggests that while the modes may be trained separately, in skilled performance, they are tightly integrated.

Ericsson’s research (2008) on deliberate practice likewise supports our approach. To become an expert in an integrated practice (like Dewey’s “inquiry”) requires the deliberate breaking down of the whole into constituent parts, training these parts, then reintegrating them into a new whole. Like a chess player practicing openings, training a single skill is a temporary and instrumental means to improving a later integrated strategy.

### Relations and processes over objects and states

Our third principle asserts, as in Dewey’s “inquiry,” that experience and nature are better apprehended as dynamic, contextual processes and relationships rather than static objects or categorical states. This process-oriented view challenges our tendency to reify abstract concepts and treat them as objects, offering a more fluid understanding that reduces both intellectual rigidity and personal suffering. While dividing the world into discrete objects is instrumentally useful, these divisions are provisional.

Whitehead’s process philosophy provides a crucial foundation for this principle. He critiqued what he called the “bifurcation of nature”—the division of nature into two halves: nature as apprehended in awareness, and a separate nature which is the cause of awareness. This bifurcation, Whitehead argued, leads to “scientific materialism,” which treats the universe as made up of distinct, isolated objects that interact mechanistically, separate from the world we experience. By contrast, Whitehead proposed that nature consists of processes rather than substances: at the most fundamental level, there are no separate objects—only interrelated processes (Whitehead 1978). This bifurcation critique directly addresses the subject/object split that underlies latent Platonism, revealing it as a false dualism that obscures the processual character of reality. Whitehead’s “fallacy of misplaced concreteness” (Whitehead 2010, 58)—treating abstractions as if they were concrete realities—parallels our critique of reifying concepts, and his process philosophy offers a framework for understanding reality as neither purely conceptual nor purely material, but as dynamic, relational processes. Whitehead’s idea prefigures Dewey’s critique of “misplaced” realism (1938, 515)—and Dewey cites Whitehead’s *Science and the Modern World* elsewhere in his *Logic*.

Building on Whitehead, Gregory Bateson critiqued science’s reductionist focus on matter arguing that systems are not static entities, but are composed of interconnected processes. His cybernetic theory emphasized self-regulating feedback circuits enabling self-organizing growth (Bateson 2000; Bateson 2002). Maturana and Varela (1980) expanded this with *autopoiesis*, describing how living systems continuously recreate themselves.

Buddhism suggests that regarding the psychological self as a process, rather than a discrete entity, reduces suffering. This aligns with Hume’s view that “each of us is nothing but a bundle or collection of different perceptions that follow each other enormously quickly and are in a perpetual flux and movement” (Hume 1739). Similar perspectives appear in Nietzsche (J. H. Miller 1981; Nietzsche 1968, §477), William James (1890, ch 9), Dewey (Dewey 1925), and modern cognitive theories of the decentralized mind (Dennett 1993; Minsky 1986). Notably, Minsky’s “society of mind” describes cognition as emerging from decentralized processes. This view has inspired effective therapies like Schwartz’s Internal Family Systems (Schwartz and Sweezy 2020), which treats individuals as having multiple “parts” with competing motivations, and which shows promise in treating PTSD and other intractable conditions (Hodgdon et al. 2022). Though these approaches differ in many other regards, their opposition to the self as a static entity demonstrates how shifting from a fixed self-concept to a fluid process can reduce suffering.

In *Neither/Nor*, we apply this “society of mind” thinking at three levels: first, as individuals, we are multiplicities. Second, these collaborative and conflicting parts originate through social interaction and cultural learning, but these very dynamics produce both benefits in moderating our behavior (Elias 2000; Freud 2001; Vygotsky 2012) and potential pathologies. Third, these same dynamics emerge within group conflicts, so internal conflicts can illuminate external ones and vice versa.

In contrast to mechanistic approaches that freeze moments and isolate phenomena from their temporal and historical context, *Neither/Nor* suggests skills for evaluating the self, scientific progress, and society at the process and relational level, with the capacity to change frames when necessary. This emphasis on process over substance reveals a broader cultural bias toward abstract, static categories over dynamic processes (McGilchrist 2009; McGilchrist 2021). While empirically determined categories improve prediction, there is a danger of fetishizing categorization itself. Our need for categorical certainty can lead to a retreat into abstraction, increasing rigidity and suffering, and creating impasses across philosophical and scientific domains. This is precisely why we return to the diabetes example: it makes the stakes concrete. Viewing blood sugar as a dynamic process rather than a numerical quantity exemplifies thinking in terms of relations and processes rather than fixed states.

Traditional psychiatric models often conceptualize depression as a discrete diagnostic entity—a latent disease state that one either has or does not have, analogous to somatic conditions like a fractured limb. However, network theory in psychopathology (Borsboom 2017; Cramer et al. 2010; Granic and Hollenstein 2003) reconceptualizes depression. Rather than treating it as an object-like disease entity, which we argue contains latent Platonist assumptions, such approaches treat depression as an emergent phenomenon arising from dynamic, self-reinforcing interactions between symptoms. For example, insomnia may lead to fatigue, which reduces social engagement, thereby increasing loneliness and negatively affecting mood, which in turn exacerbates sleep disturbance. Wichers et al. (2015) demonstrated that identifying the specific symptom-symptom feedback loops active in an individual provides greater predictive power for relapse than aggregated severity scores—suggesting that the pattern of interactions matters more than their summed intensity. This shift from categorical to processual models has direct therapeutic implications. Process-based cognitive behavioral therapy (Hayes et al. 2006) intervenes in feedback systems directly rather than treating depression as a singular pathological state. This moves away from treating depression as a singular “thing” and toward shifting maladaptive process dynamics.

This reconceptualization also aligns more closely with lived experience. Depressive states often feel like being trapped in a self-reinforcing pattern rather than possessing a discrete entity—what Ratcliffe (Ratcliffe 2015) describes as alterations in “existential feelings” that shape how possibilities appear. When someone reports “I can’t see a way out,” they frequently describe precisely this phenomenology of a vicious cycle in which each symptom forecloses options. Network theory thus offers not only improved predictive validity but a framework more adequate to both the mechanisms and phenomenology of depressive experience—yielding a more precise, adaptive, and compassionate approach to scientific inquiry and clinical intervention.

### Trial-and-error learning

*Neither/Nor* advocates for practical experimentation through circumscribed trials. “Trial-and-error” itself embodies our philosophy—trials take place in experience, while assessing errors requires conceptual judgment, creating a natural oscillation between skills. When we engage in deliberate, time-bound experimentation, failure becomes valuable information rather than wasted effort (Cole 2014; Creely et al. 2019).

Expertise ultimately relies on “unreflective situational responses” (Dreyfus and Dreyfus 2005) developed through repeated iteration rather than rational reflection alone. This approach trains intuition through non-linear improvisation in experience (Sawyer 2021). The deliberate practice of facing challenging situations repeatedly, coupled with collaborative evaluation of outcomes, creates a social dimension to trial-and-error learning that accelerates the development of expertise.

Yet emphasizing trial-and-error does not mean discarding inherited wisdom. The oscillation principle applies here too: while favoring direct experience, we value conceptual knowledge that has emerged from generations of practice. Cultural evolutionary research demonstrates that humans learn powerfully through “cumulative culture” (Henrich and Muthukrishna 2021; Muthukrishna 2023)—group-specific knowledge, practices, and technologies refined over generations. These “cultural packages” form the basis of cumulative culture, allowing subsequent generations to avoid, e.g., testing every mushroom for toxicity. This creates a foundation and ratcheting effect from which further trial-and-error experimentation can proceed.

When cultural packages conflict—as in the tension between hierarchical supremacy and pluralistic democracy—our approach does not seek reconciliation between incompatible positions. Rather than attempting to mediate between fundamentally opposed frameworks that cannot both be true, reconstruction becomes necessary (Solymosi 2025). The criterion for evaluation is not “which tradition is right” but “what works to reduce suffering and enable flourishing” when tested in experience. This requires oscillation between understanding the historical context and social functions of competing cultural packages (the conceptual skill) and observing their actual consequences in practice (the experiential skill). Through this process, we can reconstruct new approaches that learn from what works in each tradition while transcending their incompatibilities, rather than seeking an illusory harmony between positions that cannot be reconciled.

This process parallels Darwin’s understanding of variation (not just in traits, but within experience) and selection (not just whether an organism survives, but as judging success and failure), reflecting how species evolve (Darwin 2008). The management of Type 1 diabetes exemplifies this principle perfectly: while the conceptual facts are indispensable (carbohydrates raise blood sugar, insulin reduces it), effective management requires continuous experimentation to calibrate action to context, with each meal and insulin dose becoming a trial that trains intuition through feedback.

Children benefit most not from immediate correction or perfection, but from the freedom to experiment. Treiman’s research shows that when children are encouraged to use phonetic or “invented” spelling, their spellings reveal and support growing phonological awareness and literacy, rather than simply reflecting random mistakes (Bruck and Treiman 1990; Treiman 2000). These so-called errors are not random; they reflect how children systematically apply and test sound-letter rules. Similarly, Schmidt and Bjork’s (1992) motor learning research shows that making mistakes under “desirable difficulty” conditions (comparable to Vygotsky’s “zone of proximal development”; Vygotsky 1978) leads to better retention and generalizability than error-free repetition. Together, these examples demonstrate our fourth principle: learning becomes robust when students are allowed to engage in iterative, trial-and-error experimentation, not pushed toward premature accuracy.

### Conditional historicism over linear causality/prediction

*“Lack of historical sense is the family failing of all philosophers…”* (Nietzsche 1996, 13)

*“All narratives of scientific development start in midstream, with the scientific process already underway.”* (Kuhn 2022a, 7)

In lectures in the 1980s, the philosopher of science Thomas Kuhn explicitly distinguished between two approaches to history (Kuhn 2022b). What we term “present-focused” history—exemplified by science textbooks—treats past developments as steps toward our current understanding. In his unfinished book in the 1990s, Kuhn (2022c) argued that the “present-focused” lens can distort past scientific frameworks. Though Dewey earlier emphasized historicism, arguing that logical forms and methods change over history (1938), we choose to use Kuhn because he explicitly distinguishes between “past-focused” and “present-focused” types of history, providing a historicist framework that aligns with our two skills.

When we say “The Ancient Greeks wrongly believed the sun was a planet,” we anachronistically impose our concept of “planet” onto Greek cosmology. The Greek term *planētēs* (“wandering star”) simply referred to celestial bodies that moved relative to “fixed stars.” Within this framework, classifying the sun as *planētēs* wasn’t an error but a tautological truth: the sun obviously moved. This recontextualization reveals that the Greeks weren’t misguided but operated within a different, internally coherent conceptual system, with different priorities.

From the “present-focused” historical perspective, the Greeks’ classification of the sun as *planētēs* represents an understandable error on the path to modern astronomical understanding. “Past-focused” history, by contrast, attempts to understand historical frameworks on their own terms, revealing their internal coherence and contingent development. From this view, the Greeks weren’t wrong—they operated within a different conceptual system where classifying the sun as a “fixed star” would be nonsensical since it visibly moved across the sky.

Just as scientific paradigms function well when rigid, until accumulating anomalies force the paradigm itself to change, individuals can become entrenched in personal conceptual frameworks (personal narratives, fixed identities, diagnoses, political positions) which may no longer help them navigate new experience; the resulting tension can result in an “identity crisis” which exposes the limits of the existing model. Understanding Kuhn this way allows his historical analysis to illuminate individual psychology: both scientists and individuals regain flexibility not by adding to an old framework but by recognizing its contingencies across experiences and contexts and experimenting with alternatives. Historicism, then, becomes a technique which improves flexibility.

We now argue that concepts themselves (and by extension “thinking”) have sociohistorical origins. We further argue that investigating these origins through “historicizing” is a key tool for producing flexibility. These developments can be traced through both individual development and species evolution. This just means that our capacities for, e.g., speech, upright walking, cooperation, and abstraction develop within the life of each individual (ontogeny), but they also emerged in the evolution of our species (phylogeny). Although these two developmental timescales illuminate each other, we do not claim that ontogeny straightforwardly recapitulates phylogeny.

Our fifth principle thus states that all human endeavors are contingent products of sociohistorical evolution (Elias 2000; Fleck 2012; Kuhn 2022a; Longino 1990; Nietzsche 2013; Polanyi 1962). These develop through processes analogous to Darwin’s theory: variation, selection, and retention shaped by contextual pressures rather than teleological ends (Heyes 2018). Kuhn (2012) extended Darwin’s perspective to the history of science, showing that progress occurs through revolutionary shifts starting *from* simple beginnings, not *towards* predetermined ends.

Crucially, both Darwin and Kuhn examined history rather than present forms, discovering that current structures can be best understood through their historical development. Rather than attempting to understand the past as a teleological step towards the present, Darwin and Kuhn attempted to understand the past as contingent, but coherent in its own right. We argue that this historicist approach was what led them to their insights, and we extend this approach to all cognitive capacities and sociohistorical developments. Even consciousness itself (Marx et al. 2014; Nelson 1996; Nietzsche 2001; Tomasello 2021) and reason (Durkheim and Mauss 2009; Dutilh Novaes 2020; Heyes 2018; Luria 1976; Vygotsky 2012) can be understood as the product of contingent social history.

We assert that, because it makes the present the conceptual goal of the past, Kuhn’s “present-focused” history corresponds to our conceptual skill. Because it makes the past into a complex but experientially coherent whole, Kuhn’s “past-focused” history corresponds to our experiential skill.

At the individual level, healthy inquiry depends on the same oscillation: the capacity to move between a conceptual personal narrative and experiential re-entry into how earlier moments actually felt. The conceptual skill alone risks forcing the past into a narrative that serves the present self (“I moved to London to meet my partner”); the experiential skill alone risks ruminating on past states. Oscillation allows each to challenge and correct the other, making narratives flexible.

To illustrate our analogy between individual experience and history, we use an example from psychotherapy: A historical examination of an individual’s past (as in psychoanalysis) can make the present self malleable for new patterns to emerge. When combined with present-focused conceptual approaches (e.g., CBT), the new patterns can be proactively defined. The failure mode of psychoanalysis—endless investigation of the past—and the failure mode of CBT—attempting to overwrite current patterns without engaging with past conditioning—also illustrate how our oscillation approach can work in therapeutic interventions. First access past experiences to increase flexibility, then lay down new conceptual structures (Ecker et al. 2024).

In this sense, oscillation is not merely an abstract epistemic principle but a trainable personal skill. We argue that this capacity underwrites both individual transformation and collectively-fueled paradigm change.

Buddhism, too, provides a way to trace backwards developmentally to introduce flexibility, and to allow a new way of thinking and acting in the world to emerge. Psychotherapeutic interventions which target trauma, we argue, follow a similar pattern. Kuhn’s own method (2012), as well as Darwin’s (2008), show that even the sciences can be changed through the tracing of historical origins.

### Implications for reducing suffering and learning to flourish

At its core, *Neither/Nor* is a response to a specific way suffering arises in contemporary life. We call the underlying pattern latent Platonism: the conviction (mostly implicit, as the term suggests) that our concepts are more real than our experience, that the world should conform to our fixed conceptual models, and that if it does not, something is wrong with us, other people, or the world. Young people absorb this stance early. They learn to cling to fixed categories like success and failure, attractive and ugly, worthy and unworthy. Inevitably, they encounter tensions and collisions when these categories meet lived experience. For example, identity development research shows that adolescents often prematurely commit to rigid self-concepts—what Marcia termed “identity foreclosure”—adopting fixed categories about who they are without exploration (for meta-analysis, see Kroger et al. 2010).

Blood sugar inexplicably fails to follow the formulas given to diabetics, grief interrupts plans, the dream job or partner fails to solve all problems. This structural mismatch between rigid concepts and lived experience generates suffering as an inevitable consequence; flourishing requires reversing this process by holding concepts lightly and trusting experience as legitimate data.

Instead of revising the concepts, latent Platonism results in us blaming ourselves or attacking others for not conforming to our concepts. Rumination, self-blame, and tightening control attempts may address the craving for certainty in the short-term while increasing intolerance to uncertainty in the long-term. The result is anxiety, depression, obsessive-compulsive disorder (OCD), addiction of all sorts, loneliness, and a gradual shrinking of agency. Some double down on the same failing but reassuringly rigid framework. Others flip from one framework to another (e.g., from hardcore secularism to moralistic dogmatism, or across political poles), but the underlying pattern of suffering persists.

This is where flourishing enters. Here we are broadly aligned with pragmatist and experiential theories of education, from Dewey’s account of inquiry and growth (Dewey 1916b; 1938), through Bruner’s attention to narrative (1990; 1986), to Vygotsky’s scaffolding in the “zone of proximal development” (1978; 2012). However, *Neither/Nor* puts special emphasis on how learners relate these phases to one another. When concepts are held lightly, oscillated with experience as a source of legitimate data, people gain adaptive flexibility. They shift from a discovery stance, in which identity, purpose, and meaning are treated as mind-independent facts that must be correctly categorized, to a creative stance, in which these are understood as being co-authored through action in the social world. This does not mean that everything is created. It means that questions such as “What do I value?”, “What kind of person do I wish to become?” and “What contribution feels worth making?” can be engaged experimentally in lived contexts, rather than blocked by unconscious metaphysical commitments which require certainty.

In educational practice, the implication is that we can design developmentally sensitive programs that proceed systematically, where learners first encounter the limits of rigid conceptual frameworks in safe, bounded ways, then practice testing those frameworks within their direct experience. Through reflection, the outcomes of those experiences can be flexibly updated and used provisionally in further iterations. Experiences with a wide diversity of social and environmental contexts are probably optimal. As examples, wilderness immersion, project-based learning, dialogical narrative practices, and contemplative or mindfulness exercises can all be tried, holding an intention to oscillate between concept and experience, and to experiment (Granic 2025). The goal is not only to reduce suffering, but to cultivate the conditions for grappling with uncertainty across domains. In that sense, *Neither/Nor* is offered as an “educational art” rather than “uniform rules of procedure” (Dewey 1929b, 13–14): a way of structuring experience so that the same capacities which once intensified suffering become the basis for a more flexible, resilient, iterative process of oscillation.

## Conclusion

The distinctive contribution of *Neither/Nor* lies not in any single insight but in three operational moves absent from prior syntheses: (1) the insistence on isolated training of each skill before integration—unlike “Both/And” approaches that combine skills immediately, we argue that facility with negation (the capacity to interrupt and oscillate one’s current mode) requires first developing each skill independently; (2) the explicit inclusion of negation as a trainable component rather than merely a logical operation—this capacity to actively suspend conceptual analysis or interrupt habitual action must be deliberately practiced; and (3) the simultaneous application of this framework to individual suffering, disciplinary inquiry, and therapeutic intervention, demonstrating that the same oscillation principles operate across scales. While pragmatists like Dewey described the unity of intelligent inquiry, and process philosophers like Whitehead described reality’s processual nature, *Neither/Nor* operationalizes the training regime that develops the capacity for this unity and process-awareness, rendering philosophical insights actionable.

In this paper, we had three objectives: to present *Neither/Nor* as a pragmatic framework, to discuss its lineages, and to articulate its central principles. At its core, *Neither/Nor* asserts that conceptual thinking and experiential knowledge function as complementary skills that can be deliberately trained and oscillated. Neither theory nor practice alone will suffice—both are required. We argue that this oscillation between these two skills happens frequently and unconsciously, but that deliberate training can enhance awareness and aptitude of the two skills.

Our framework provides a methodology for navigating between concepts and experience, enabling dynamic change at multiple scales. We argue that the same principles of change underlie all three levels—intrapersonal, interpersonal, and international—providing not only a methodology for enacting these changes but also criteria for assessing change strategies. This scalability makes *Neither/Nor* powerful as both a philosophical framework and a practical approach to transformation.

While *Neither/Nor* draws from philosophical traditions across millennia—from Buddhism to contemporary cognitive science—it makes a contribution by operationalizing these philosophical positions into trainable skills. Thinkers like Zhuangzi, the Ancient Greek Pyrrhonists, Nietzsche, Wittgenstein, Deleuze, Bateson, and McGilchrist have brilliantly drawn attention to the limitations of rigid conceptual thinking, but their insights remain notoriously difficult to implement. *Neither/Nor* asserts that underlying these diverse thinkers is a technique whose transformational potential makes it both paradoxical to articulate and challenging to practice.

The five principles of *Neither/Nor* offer promising applications across domains. However, we acknowledge several limitations: First, while central to our framework, the oscillation between skills lacks practical validation. Although we have reviewed some of the extant data that supports our framework, it seems important for future research to directly test the main premises of *Neither/Nor* through intervention designs (e.g., in education contexts) that can track the efficacy of oscillation practices with properly controlled studies (e.g., Granic 2025). Second, our synthesis of Eastern and Western traditions necessarily condenses nuanced ideas; a more extensive exploration is planned for a forthcoming book. Third, detailed guidance on implementing these principles—particularly training methods for oscillation—could not be fully elaborated here. Fourth, we could not engage with potential critiques from competing philosophical traditions. Finally, while we addressed personal suffering, space constraints limited our exploration of broader societal implications. Future work through further publications, interdisciplinary collaboration, and empirical validation will be essential for refining and extending the *Neither/Nor* approach.

## Data Availability

No datasets were generated or analyzed during the current study.

## AI usage

Anthropic Claude 3.7 Sonnet was used in manuscript preparation between 18 February 2025 and 14 April 2025 to help remove redundancy and reduce the length of this paper. Anthropic Claude 4.5 Sonnet was used in manuscript preparation between 24 November 2025 and 15 December 2025 to track changes between revisions, and to confirm that we had dealt with reviewers’ comments.

## References

[CR1] Open Science Collaboration (2015) Estimating the reproducibility of psychological science. Science 349(6251):aac4716. 10.1126/science.aac4716 10.1126/science.aac4716 26315443

[CR2] Peirce Charles S (1878) How to make our ideas clear. Pop Sci Mon 1878:286–302. 12

[CR3] Alcoff L (2006) Visible identities: race, gender, and the self. Oxford University Press, Oxford. 10.1093/0195137345.001.0001

[CR4] Althusser L (1971) Ideology and ideological state apparatuses (notes towards an investigation). In: Lenin and philosophy, and other essays (trans: Brewster B). New Left Books, London, pp 127–186

[CR5] Aquinas T (1920) Summa theologica (trans: Fathers of the English Dominican Province), Second and Revised edn. Christian Classics, Westminster

[CR6] Aristotle (2016) De anima (trans: CJ Shields). Clarendon, Oxford

[CR7] Atwoli L, Baqui AH, Benfield T et al(2021) Call for emergency action to limit global temperature increases, restore biodiversity, and protect health Lancet 398(10304):939–941. 10.1016/S0140-6736(21)01915-2 34496267 10.1016/S0140-6736(21)01915-2 PMC8428481

[CR8] Ayer AJ (1979) The construction of our theory of the physical world. In: Honderich T, Burnyeat M (eds) Philosophy as it is. Penguin, Harmondsworth, pp 311–348

[CR9] Baillargeon R (1987) Object permanence in 3½- and 4½-month-old infants. Dev Psychol 23(5):655–664. 10.1037/0012-1649.23.5.655

[CR10] Bateson G (2000) Steps to an ecology of mind. University of Chicago Press, Chicago

[CR11] Bateson G (2002) Mind and nature: a necessary unity. Hampton Press, New York

[CR12] Beale J, Kidd IJ (2017) Wittgenstein and scientism. Routledge, London

[CR13] Beck AT, Rush AJ, Shaw BF et al. (2024) Cognitive therapy of depression, 2nd edn. Guilford Press, New York

[CR14] von Bertalanffy L (2015) General system theory: foundations, development, applications, Revised edn. George Braziller, Inc, New York

[CR15] Blumenthal HJ, Armstrong AH (2024) Platonism. In: Encyclopedia Britannica, December 2024. Accessed 24 Feb 2026

[CR16] Bodhi B trans (2000) The connected discourses of the Buddha: a translation of the Samyutta Nikāya. Wisdom Publications, Boston

[CR17] Bodhi B trans (2017) The Suttanipāta: an ancient collection of the Buddha’s discourses; together with its commentary, elucidator of the supreme meaning, Paramatthajotikā II, and excerpts from the Niddesa. Wisdom Publications, Somerville

[CR18] Borsboom D (2017) A network theory of mental disorders. World Psychiatry 16(1):5–13. 10.1002/wps.20375 10.1002/wps.20375 PMC5269502 28127906

[CR19] Brosschot JF, Pieper S, Thayer JF (2005) Expanding stress theory: prolonged activation and perseverative cognition. Psychoneuroendocrinology 30(10):1043–1049. 10.1016/j.psyneuen.2005.04.008. November 2005 15939546 10.1016/j.psyneuen.2005.04.008

[CR20] Brozović D (2023) Societal collapse: a literature review. Futures 145:103075. 10.1016/j.futures.2022.103075

[CR21] Bruck M, Treiman R (1990) Phonological awareness and spelling in normal children and dyslexics: the case of initial consonant clusters. J Exp Child Psychol 50(1):156–178. 10.1016/0022-0965(90)90037-9 10.1016/0022-0965(90)90037-9 2398331

[CR22] Bruner J (1986) Actual minds, possible worlds. Harvard University Press, Cambridge

[CR23] Bruner J (1990) Acts of meaning: four lectures on mind and culture. Harvard University Press, Cambridge

[CR24] Call J, Tomasello M (2008) Does the chimpanzee have a theory of mind? 30 years later. Trends Cogn Sci 12(5):187–192. 10.1016/j.tics.2008.02.010. May 2008 18424224 10.1016/j.tics.2008.02.010

[CR25] Chalmers DJ (1996) The conscious mind: in search of a fundamental theory. Oxford University Press, New York

[CR26] Cohen HF (2015) The rise of modern science explained: a comparative history. Cambridge University Press, Cambridge

[CR27] Cole S (2014) “Fail again. Fail better.” Failure in the creative process. AJHA 1(3):183–192. 10.30958/ajha.1-3-1

[CR28] Cramer AOJ, Waldorp LJ, Van Der Maas HLJ et al. (2010) Comorbidity: a network perspective. Behav Brain Sci 33(2–3):137–150. 10.1017/S0140525X0999156 10.1017/S0140525X09991567 20584369

[CR29] Creely E, Henderson M, Henriksen D (2019) Failing to succeed: the value of failure in creativity. In: Proceedings of Society for Information Technology & Teacher Education International Conference, Las Vegas, March 2019

[CR30] Darwin C (2008) On the origin of species. Oxford University Press, New York

[CR31] David M (2022) The correspondence theory of truth. In: Zalta EN (ed) The Stanford encyclopedia of philosophy, Summer 2022 Metaphysics Research Lab, Stanford University

[CR32] Deleuze G, Guattari F (1994) What is philosophy? Columbia University Press, New York, (trans: H Tomlinson, G Burchell)

[CR33] DeLoache JS (2004) Becoming symbol-minded. Trends Cogn Sci 8(2):66–70. 10.1016/j.tics.2003.12.004 10.1016/j.tics.2003.12.004 15588810

[CR34] Dennett DC (1993) Consciousness explained. Penguin, London

[CR35] Descartes R (1996) Meditations on first philosophy: with selections from the objections and replies. Cambridge University Press, Cambridge, (trans: JG Cottingham)

[CR36] Dewey J (1916a) Essays in experimental logic. In: Boydston JA (ed) The middle works, 1899–1924, vol 10. Southern Illinois University Press, Carbondale

[CR37] Dewey J (1916b) Democracy and education. In: Boydston JA (ed) The middle works, 1899–1924, vol 9. Southern Illinois University Press, Carbondale

[CR38] Dewey J (1925) Experience and nature. In: Boydston JA (ed) The later works, 1925–1953, vol 1. Southern Illinois University Press, Carbondale

[CR39] Dewey J (1929a) The quest for certainty: a study of the relation of knowledge. In: Boydston JA (ed) The later works, 1925–1953, vol 4. Southern Illinois University Press, Carbondale

[CR40] Dewey J (1929b) The sources of a science of education. In: Boydston JA (ed) Essays, the sources of a science of education, individualism, old and new, and construction and criticism, vol 5. Southern Illinois University Pr, Carbondale, pp 1–40

[CR41] Dewey J (1938) Logic: the theory of inquiry. In: Boydston JA (ed) The later works, 1925–1953, vol 12. Southern Illinois University Press, Carbondale

[CR42] Dignāga (1968) Dignāga, on perception: being the Pratyakṣapariccheda of Dignāga’s Pramāṇasamuccaya from the Sanskrit fragments and the Tibetan versions. Harvard University Press, Cambridge, (trans: M Hattori)

[CR43] Diogenes Laertius (1959) Lives of eminent philosophers. Harvard University Press, Cambridge, volume II: books 6–10 (trans: RD Hicks)

[CR44] Dreyfus HL, Dreyfus SE (2005) Peripheral vision: expertise in real world contexts. Organ Stud 26(5):779–792. 10.1177/0170840605053102

[CR45] Duns Scotus J (2024) Ordinatio II d.1-3 volume seven of the critical edition. On creation, and the angels. https://www.aristotelophile.com/Books/Translations/ScotusOrdinatio2dd.1-3.pdf. Accessed 18 Apr 2025

[CR46] Durkheim É, Mauss M (2009) Primitive classification. Taylor & Francis, Hoboken

[CR47] Dussutour A (2021) Learning in single cell organisms. Biochem Biophys Res Commun 564:92–102. 10.1016/j.bbrc.2021.02.018. July 2021 33632547 10.1016/j.bbrc.2021.02.018

[CR48] Dutilh Novaes C (2020) The dialogical roots of deduction: historical, cognitive, and philosophical perspectives on reasoning. Cambridge University Press, Cambridge. 10.1017/9781108800792

[CR49] Dweck C (2016) Mindset: the new psychology of success: how we can learn to fulfill our potential: parenting, business, school, relationships. Random House, New York, Updated edition

[CR50] Ecker B, Ticic R, Hulley L (2024) Unlocking the emotional brain: memory reconsolidation and the psychotherapy of transformational change, 2nd edn. Routledge, New York

[CR51] Eckersley R (2006) Is modern Western culture a health hazard? Int J Epidemiol 35(2):252–258. 10.1093/ije/dyi235 10.1093/ije/dyi235 16303804

[CR52] Elias N (2000) The civilizing process: sociogenetic and psychogenetic investigations. Dunning E, Goudsblom J, Mennell S (eds). Blackwell, Oxford

[CR53] Ericsson KA (2008) Deliberate practice and acquisition of expert performance: a general overview. Acad Emerg Med 15(11):988–994. 10.1111/j.1553-2712.2008.00227.x. November 2008 18778378 10.1111/j.1553-2712.2008.00227.x

[CR54] Ernst M, Niederer D, Werner AM et al. (2022) Loneliness before and during the COVID-19 pandemic: a systematic review with meta-analysis. Am Psychol 77(5):660–677. 10.1037/amp0001005 10.1037/amp0001005 PMC9768682 35533109

[CR55] Eva KW (2005) What every teacher needs to know about clinical reasoning. Med Educ 39(1):98–106. 10.1111/j.1365-2929.2004.01972.x 10.1111/j.1365-2929.2004.01972.x 15612906

[CR56] Fleck L (2012) Genesis and development of a scientific fact. University of Chicago Press, Chicago, (trans: F Bradley, TJ Trenn)

[CR57] Freud S (2001) Totem and taboo: some points of agreement between the mental lives of savages and neurotics. Routledge, London, (trans: J Strachey)

[CR58] Glock H-J (2000) Animals, thoughts and concepts. Synthese 123(1):35–64. 10.1023/A:1005295521736

[CR59] Gore FM, Bloem PJ, Patton GC et al. (2011) Global burden of disease in young people aged 10–24 years: a systematic analysis. Lancet 377(9783):2093–2102. 10.1016/S0140-6736(11)60512-6 10.1016/S0140-6736(11)60512-6 21652063

[CR60] Goyal M, Singh S, Sibinga EMS et al. (2014) Meditation programs for psychological stress and well-being: a systematic review and meta-analysis. JAMA Intern Med 174(3):357. 10.1001/jamainternmed.2013.13018 10.1001/jamainternmed.2013.13018 PMC4142584 24395196

[CR61] Granic I, Hollenstein T (2003) Dynamic systems methods for models of developmental psychopathology. Dev Psychopathol 15(3):641–669. 10.1017/S0954579403000324 10.1017/s0954579403000324 14582935

[CR62] Granic I (2025) Liminal learning: new social and developmental practices for emerging adults. Paper presented at the Harvard Graduate School of Education’s human transformation in a time of metacrisis, Boston, May 2025

[CR63] Grant E (2008) The foundations of modern science in the Middle Ages: their religious, institutional, and intellectual contexts. Cambridge University Press, Cambridge

[CR64] Hadot P (1995) Philosophy as a way of life: spiritual exercises from Socrates to Foucault (trans: M Chase). Davidson AI (ed). Blackwell, Malden

[CR65] Hahn D (2019) Friendly feud: how best to study the physical properties of the universe. Boston University, 2019. https://www.bu.edu/articles/2019/friendly-physics-feud/. Accessed 15 Apr 2025

[CR66] Hardy GH (2019) A mathematician’s apology. Cambridge University Press, Cambridge

[CR67] Hayes SC, Luoma JB, Bond FW et al. (2006) Acceptance and commitment therapy: model, processes and outcomes. Behav Res Ther 44(1):1–25. 10.1016/j.brat.2005.06.006 10.1016/j.brat.2005.06.006 16300724

[CR68] Heidegger M (1997) Kant and the problem of metaphysics. Indiana University Press, Bloomington, (trans: R Taft)

[CR69] Henrich J, Muthukrishna M (2021) The origins and psychology of human cooperation. Annu Rev Psychol 72(1):207–240. 10.1146/annurev-psych-081920-042106 10.1146/annurev-psych-081920-042106 33006924

[CR70] Heyes C (2018) Cognitive gadgets: the cultural evolution of thinking. Belknap Press, Cambridge

[CR71] Hodgdon HB, Anderson FG, Southwell E et al. (2022) Internal family systems (IFS) therapy for posttraumatic stress disorder (PTSD) among survivors of multiple childhood trauma: a pilot effectiveness study. J Aggress Maltreat Trauma 31(1):22–43. 10.1080/10926771.2021.2013375

[CR72] Hume D (1739) A treatise of human nature: a critical edition. Clarendon Press, Oxford

[CR73] Hume D (1766) An enquiry concerning human understanding: a critical edition. Clarendon Press, Oxford

[CR74] Iyengar S, Lelkes Y, Levendusky M et al. (2019) The origins and consequences of affective polarization in the United States. Annu Rev Polit Sci 22:129–146. 10.1146/annurev-polisci-051117-073034

[CR75] Jacobi FH (1995) Jacobi to Fichte (1799). In: Main philosophical writings and the novel Allwill McGill-Queen’s University Press, Montréal, pp 497–536. 10.1515/9780773564121

[CR76] James W (1884) What is an emotion? Mind 9(34):188–205. 10.1093/mind/os-IX.34.188

[CR77] James W (1890) The principles of psychology. Henry Holt and Company, New York

[CR78] James W (1907) Lecture VI: pragmatism’s conception of truth. In: Pragmatism: a new name for some old ways of thinking Longmans, New York, pp 197–236

[CR79] Kant I (1998) Critique of pure reason. Cambridge University Press, Cambridge, (trans: P Guyer, AW Wood)

[CR80] Kashdan TB, Rottenberg J (2010) Psychological flexibility as a fundamental aspect of health. Clin Psychol Rev 30(7):865–878. 10.1016/j.cpr.2010.03.001. November 2010 21151705 10.1016/j.cpr.2010.03.001 PMC2998793

[CR81] Kasser T (2016) Materialistic values and goals. Annu Rev Psychol 67(1):489–514. 10.1146/annurev-psych-122414-033344 10.1146/annurev-psych-122414-033344 26273896

[CR82] Kazdin AE (2008) Evidence-based treatment and practice: new opportunities to bridge clinical research and practice, enhance the knowledge base, and improve patient care. Am Psychol 63(3):146–159. 10.1037/0003-066X.63.3.146 10.1037/0003-066X.63.3.146 18377105

[CR83] Khoury B, Lecomte T, Fortin G et al. (2013) Mindfulness-based therapy: a comprehensive meta-analysis. Clin Psychol Rev 33(6):763–771. 10.1016/j.cpr.2013.05.005. August 2013 23796855 10.1016/j.cpr.2013.05.005

[CR84] Kroger J, Martinussen M, Marcia JE (2010) Identity status change during adolescence and young adulthood: a meta-analysis. J Adolesc 33(5):683–698. 10.1016/j.adolescence.2009.11.002 10.1016/j.adolescence.2009.11.002 20004962

[CR85] Kuhn TS (2012) The structure of scientific revolutions. University of Chicago Press, Chicago

[CR86] Kuhn TS (2022a) Scientific knowledge as historical product. In: Mladenović B (ed) The last writings of Thomas S. Kuhn: incommensurability in science. University of Chicago Press, Chicago, pp 1–19

[CR87] Kuhn TS (2022b) The presence of past science (the Shearman Memorial Lectures). In: Mladenović B (ed) The last writings of Thomas S. Kuhn: incommensurability in science. University of Chicago Press, Chicago, pp 23–71

[CR88] Kuhn TS (2022c) The plurality of worlds: an evolutionary theory of scientific development. In: Mladenović B (ed) The last writings of Thomas S. Kuhn: incommensurability in science. University of Chicago Press, Chicago, pp 103–266

[CR89] Lang PJ (1994) The varieties of emotional experience: a meditation on James-Lange theory. Psychol Rev 101(2):211–221. 10.1037/0033-295x.101.2.211 10.1037/0033-295x.101.2.211 8022956

[CR90] Levin ME, Hildebrandt MJ, Lillis J et al. (2012) The impact of treatment components suggested by the psychological flexibility model: a meta-analysis of laboratory-based component studies. Behav Ther 43(4):741–756. 10.1016/j.beth.2012.05.003. December 2012 23046777 10.1016/j.beth.2012.05.003

[CR91] Longino HE (1990) Science as social knowledge: values and objectivity in scientific inquiry. Princeton University Press, Princeton

[CR92] Luria AR (1976) Cognitive development: its cultural and social foundations. Harvard University Press, Cambridge, (trans: M Lopez-Morillas)

[CR93] Lyon P (2015) The cognitive cell: bacterial behavior reconsidered. Front Microbiol April 2015:6. 10.3389/fmicb.2015.00264 10.3389/fmicb.2015.00264 PMC4396460 25926819

[CR94] Mamede S, van Gog T, van den Berge K et al. (2010) Effect of availability bias and reflective reasoning on diagnostic accuracy among internal medicine residents. JAMA 304(11):1198–1203. 10.1001/jama.2010.1276. September 2010 20841533 10.1001/jama.2010.1276

[CR95] Marx K, Engels F (2014) Rough notes, formerly known as “I. Feuerbach,” drawn from “the German Ideology” manuscripts. In: Carver T, Blank D (eds) Marx and Engels's "German Ideology" manuscripts: Presentation and analysis of the "Feuerbach chapter". Palgrave Macmillan, New York, pp 34–382

[CR96] Maturana HR, Varela FJ (1980) Autopoiesis and cognition: the realization of the living. D Reidel Publishing Company, Dordrecht

[CR97] McGilchrist I (2009) The master and his emissary: the divided brain and the making of the Western world. Yale University Press, New Haven

[CR98] McGilchrist I (2021) The matter with things: our brains, our delusions and the unmaking of the world. Perspectiva Press, London

[CR99] McLaughlin KA, Nolen-Hoeksema S (2011) Rumination as a transdiagnostic factor in depression and anxiety. Behav Res Ther 49(3):186–193. 10.1016/j.brat.2010.12.006 10.1016/j.brat.2010.12.006 PMC3042543 21238951

[CR100] Miller JH (1981) The disarticulation of the self in Nietzsche. Monist 64(2):247–261. 10.5840/monist198164217

[CR101] Miller A (2024) Realism. In: Zalta EN, Nodelman U (eds) The Stanford encyclopedia of philosophy, Summer 2024 Metaphysics Research Lab, Stanford University

[CR102] Mills CW (1997) The racial contract. Cornell University Press, Ithaca

[CR103] Minsky M (1986) The society of mind. Simon and Schuster, New York

[CR104] Murthy VH (2020) Together: loneliness, health and what happens when we find connection. Profile Books, London

[CR105] Muthukrishna M (2023) A theory of everyone: who we are, how we got here, and where we’re going. Basic Books, London

[CR106] Naghavi M (2019) Global, regional, and national burden of suicide mortality 1990 to 2016: systematic analysis for the Global Burden of Disease Study 2016. BMJ 364:l94. 10.1136/bmj.l94 10.1136/bmj.l94 PMC6598639 31339847

[CR107] Ñāṇamoli B, Bodhi B (1995) The middle length discourses of the Buddha: a translation of the Majjhima Nikāya, 3rd edn. Wisdom Publications, Boston, trans

[CR108] Ñāṇananda B (2012) Concept and reality in early Buddhist thought: an essay on papañca and papañca-saññā-saṅkhā. Dharma Grantha Mudrana Bhāraya, Sri Lanka

[CR109] Nelson K (1996) Language in cognitive development: the emergence of the mediated mind. Cambridge University Press, Cambridge. 10.1017/CBO9781139174619

[CR110] Nietzsche F (1968) The will to power. Vintage Books, New York, (trans: WA Kaufmann, RJ Hollingdale)

[CR111] Nietzsche F (1996) Human, all too human. Cambridge University Press, Cambridge, (trans: RJ Hollingdale)

[CR112] Nietzsche F (2000) The birth of tragedy. In: Basic writings of Nietzsche (trans: Kaufmann W) Random House, London, pp 1–144

[CR113] Nietzsche F (2001) The gay science: with a prelude in German rhymes and an appendix of songs. Cambridge University Press, Cambridge, (trans: J Nauckhoff, A Del Caro)

[CR114] Nietzsche F (2003) Beyond good and evil: prelude to a philosophy of the future. Penguin Books, London, (trans: RJ Hollingdale)

[CR115] Nietzsche F (2013) On the genealogy of morals: a polemic. Penguin Books, London, (trans: M Scarpitti)

[CR116] Norman GR, Monteiro SD, Sherbino J et al. (2017) The causes of errors in clinical reasoning: cognitive biases, knowledge deficits, and dual process thinking. Acad Med 92(1):23. 10.1097/ACM.0000000000001421 10.1097/ACM.0000000000001421 27782919

[CR117] Park CL (2010) Making sense of the meaning literature: an integrative review of meaning making and its effects on adjustment to stressful life events. Psychol Bull 136(2):257–301. 10.1037/a0018301 10.1037/a0018301 20192563

[CR118] Parodi KB, Holt MK, Green JG et al. (2022) Time trends and disparities in anxiety among adolescents, 2012-2018. Soc Psychiatry Psychiatr Epidemiol 57(1):127–137. 10.1007/s00127-021-02122-9 10.1007/s00127-021-02122-9 PMC8183580 34100110

[CR119] Peirce Charles Sanders (1877) The fixation of belief. Pop Sci Mon 12(November 1877):1–15

[CR120] Pihlström S (2023) Pragmatism as a mediator – seeking an illusory harmony? Contemp Pragmat 22(1):1–27. 10.1163/18758185-bja10078

[CR121] Plato (1992) Theaetetus. Hackett Publishing, Indianapolis, (trans: MJ Levett)

[CR122] Plato (1997) Republic. In: Complete works (trans: Grube GMA, Reeve rev. CDC) Hackett Publishing Company, Inc, Indianapolis, pp 971–1223

[CR123] Polanyi M (1962) The republic of science: its political and economic theory. Minerva 1(1):54–73. 1962

[CR124] Puhl RM, Heuer CA (2009) The stigma of obesity: a review and update. Obesity 17(5):941–964. 10.1038/oby.2008.636 10.1038/oby.2008.636 19165161

[CR125] Putnam H, Putnam RA (2017) Pragmatism as a way of life: the lasting legacy of William James and John Dewey. Macarthur D (ed). Belknap Press, Cambridge

[CR126] Quine WVO (1969) Epistemology naturalized. In: Ontological relativity and other essays Columbia University Press, New York, pp 69–90

[CR127] Ratcliffe M (2015) Experiences of depression: a study in phenomenology. Oxford University Press, Oxford

[CR128] Ripple WJ, Wolf C, Gregg JW et al. (2024) The 2024 state of the climate report: perilous times on planet earth. Biosci 74(12):812–824. 10.1093/biosci/biae087. October 2024

[CR129] Rizzolatti G, Arbib MA (1998) Language within our grasp. Trends Neurosci 21(5):188–194. 10.1016/S0166-2236(98)01260-0 10.1016/s0166-2236(98)01260-0 9610880

[CR130] Russell B (1911) Knowledge by acquaintance and knowledge by description. Proc Aristot Soc 11(1):108–128. 10.1093/aristotelian/11.1.108

[CR131] Ryan RM, Deci EL (2000) Self-determination theory and the facilitation of intrinsic motivation, social development, and well-being. Am Psychol 55(1):68–78. 10.1037/0003-066X.55.1.68 10.1037//0003-066x.55.1.68 11392867

[CR132] Ryan RM, Deci EL (2017) Self-determination theory: basic psychological needs in motivation, development, and wellness. Guilford Press, New York. 10.1521/978.14625/28806

[CR133] Salami M (2020) Sensuous knowledge: a black feminist approach for everyone. Bloomsbury Academic, London

[CR134] Sawyer RK (2021) The iterative and improvisational nature of the creative process. J Creat 31:100002. 10.1016/j.yjoc.2021.100002. December 2021

[CR135] Schachter S, Singer J (1962) Cognitive, social, and physiological determinants of emotional state. Psychol Rev 69(5):379–399. 10.1037/h0046234 10.1037/h0046234 14497895

[CR136] Schipper ELF, Revi A, Preston BL et al. (2022) Climate resilient development pathways. In: Pörtner H-O, Roberts DC, Tignor MMB et al. (eds) Climate change 2022: impacts, adaptation and vulnerability. Contribution of Working Group II to the Sixth Assessment Report of the Intergovernmental Panel on Climate Change Cambridge University Press, Cambridge, pp 2655–2807. 10.1017/9781009325844.027

[CR137] Schliesser E (2019) Synthetic philosophy. Biol Philos 34(2):19. 10.1007/s10539-019-9673-3

[CR138] Schmidt RA, Bjork RA (1992) New conceptualizations of practice: common principles in three paradigms suggest new concepts for training. Psychol Sci 3(4):207–218. 10.1111/j.1467-9280.1992.tb00029.x. July 1992

[CR139] Schopenhauer A (1969) The world as will and representation. Dover Publications, New York, vol 1

[CR140] Schwartz RC, Sweezy M (2020) Internal family systems therapy, 2nd edn. Guilford Press, New York

[CR141] Sellars W (1991) Empiricism and the philosophy of mind. In: Science, perception and reality Ridgeview Publishing Company, Atascadero, pp 127–196

[CR142] Sextus Empiricus (1994) Outlines of scepticism. Cambridge University Press, Cambridge

[CR143] Shorey S, Ng ED, Wong CHJ (2022) Global prevalence of depression and elevated depressive symptoms among adolescents: a systematic review and meta-analysis. Br J Clin Psychol 61(2):287–305. 10.1111/bjc.12333 10.1111/bjc.12333 34569066

[CR144] Shusterman R (2016) Practicing philosophy: pragmatism and the philosophical life. Taylor and Francis, London

[CR145] Solymosi T (2025) Neuropragmatic reconstruction, not transcendental reconciliation: a response to Pihlström’s “Pragmatism as a mediator—seeking an illusory harmony?”. Contemp Pragmat 22(1):59–79. 10.1163/18758185-bja10102

[CR146] Spinoza B (1985) The Ethics. In: Curley E (ed, trans) The collected works of Spinoza, vol 1. Princeton University Press, Princeton, pp 408–652

[CR147] Surkalim DL, Luo M, Eres R et al. (2022) The prevalence of loneliness across 113 countries: systematic review and meta-analysis. BMJ 376:e067068. 10.1136/bmj-2021-067068 10.1136/bmj-2021-067068 PMC8826180 35140066

[CR148] Tomasello M (2021) Becoming human: a theory of ontogeny. Belknap Press, Cambridge

[CR149] Treiman R (2000) The foundations of literacy. Curr Dir Psychol Sci 9(3):89–92. 10.1111/1467-8721.00067

[CR150] Tweedale M (2023) Making wonderful: ideological roots of our eco-catastrophe. University of Alberta Press, Edmonton. 10.1515/9781772126594

[CR151] Valgarðsson V, Jennings W, Stoker G et al. (2025) A crisis of political trust? Global trends in institutional trust from 1958 to 2019. Br J Political Sci 55:e15. 10.1017/S0007123424000498

[CR152] Van Norden BW (2017) Taking back philosophy: a multicultural manifesto. Columbia University Press, New York

[CR153] Vygotsky LS (1978) Mind in society: development of higher psychological processes. Cole M, Jolm-Steiner V, Scribner S et al. (eds). Harvard University Press, Cambridge

[CR154] Vygotsky LS (2012) Thought and language. Kozulin A (ed). MIT Press, Cambridge

[CR155] Vygotsky LS, Luria AR (1994) Tool and symbol in child development. In: Veer R van der, Valsiner J (eds) The Vygotsky reader by LS Vygotsky Blackwell, Oxford

[CR156] Walshe M (1995) The long discourses of the Buddha: a translation of the Digha Nikāya. Wisdom Publications, New York, trans

[CR157] Wells A (2009) Metacognitive therapy for anxiety and depression. Guilford Press, New York

[CR158] Wesselhoeft R, Sørensen MJ, Heiervang ER et al. (2013) Subthreshold depression in children and adolescents – a systematic review. J Affect Disord 151(1):7–22. 10.1016/j.jad.2013.06.010. October 2013 23856281 10.1016/j.jad.2013.06.010

[CR159] Whitehead AN (1978) Process and reality: an essay in cosmology; Gifford lectures delivered in the University of Edinburgh during the session 1927–28. Griffin DR, Sherburne DW (eds), Corrected edn. The Free Press, New York

[CR160] Whitehead AN (2010) Science and the modern world (1925), 7th edn. Free Press, New York

[CR161] Wichers M, Wigman JTW, Myin-Germeys I (2015) Micro-level affect dynamics in psychopathology viewed from complex dynamical system theory. Emot Rev 7(4):362–367. 10.1177/1754073915590623

[CR162] Wittgenstein L (2008) Philosophical investigations: the German text, with a revised English translation, 3rd edn. Blackwell, Oxford, (trans: GEM Anscombe)

[CR163] World Health Organization (2022) Mental health and COVID-19: early evidence of the pandemic’s impact: Scientific brief, 2 March 2022. World Health Organization

[CR164] Yeager DS, Dweck CS (2012) Mindsets that promote resilience: when students believe that personal characteristics can be developed. Educ Psychol 47(4):302–314. 10.1080/00461520.2012.722805. October 2012

